# Determinants and predictive model of failure of surgical repair of obstetric vesico-vaginal fistula in the Democratic Republic of the Congo

**DOI:** 10.1186/s12978-024-01779-0

**Published:** 2024-04-01

**Authors:** Justin Lussy Paluku, Barthelemy Kasi Aksanti, William C. Clemmer, Cathy Mufungizi Furaha, Eugénie Mukekulu Kamabu, Jonathan M. L. Kasereka, Benjamin Kambale Kalole, Olivier Mukuku, Zacharie Kibendelwa Tsongo, Stanis Okitotsho Wembonyama, Charles Wembonyama Mpoy, Jeannot Sihalikyolo Juakali

**Affiliations:** 1 Department of Obstetrics and Gynecology, Faculty of Medicine, University of Goma, Goma, Democratic Republic of the Congo; 2 Department of Obstetrics and Gynecology, HEAL Africa Hospital, Goma, Democratic Republic of the Congo; 3 Department of Family Medicine, American Academy of Family Physicians, Orono, USA; 4 Department of Internal Medicine, HEAL Africa Hospital, Goma, Democratic Republic of the Congo; 5 Department of Orthopedics and Trauma, HEAL Africa Hospital, Goma, Democratic Republic of the Congo; 6 Department of Maternal and Child Health, Institut Supérieur des Techniques Médicales, Lubumbashi, Democratic Republic of the Congo; 7 Department of Internal Medicine, Faculty of Medicine, University of Kisangani, Kisangani, Democratic Republic of the Congo; 8 Departments of Pediatrics and Public Health, Faculty of Medicine, University of Lubumbashi, Lubumbashi, Democratic Republic of the Congo; 9 Department of Obstetrics and Gynecology, Faculty of Medicine, University of Lubumbashi, Lubumbashi, Democratic Republic of the Congo; 10 Department of Obstetrics and Gynecology, Faculty of Medicine, University of Kisangani, Kisangani, Democratic Republic of the Congo

**Keywords:** Obstetric vesico-vaginal fistula, Predictive score, Surgical repair, DRC

## Abstract

**Introduction:**

Surgical repair of obstetric fistula aims to restore the anatomical and functional integrity of the urinary tract, enabling affected women to regain their dignity and quality of life. However, such repairs can end in a failure. The aim of this study is to develop a predictive score to identify factors influencing failure of surgical repair of obstetric vesico-vaginal fistula (FSROVVF) in the Democratic Republic of the Congo.

**Methods:**

This was an analytical cross-sectional study of 318 women with obstetric vesico-vaginal fistula (OVVF) who had undergone surgical management. A bivariate and then a multivariate analysis were performed. Score discrimination was assessed using the ROC curve and C-index, and score calibration using the Hosmer–Lemeshow test.

**Results:**

Surgical repair of OVVF was unsuccessful in 16.98% of cases (54/318). After logistic modeling, six criteria emerged as predictive factors for FSROVVF: the presence of fibrosis (AOR = 5.01; 95% CI:1.73–14.49), the presence of 2 or more fistulas (AOR = 9.04; 95% CI:3.01–27.13), the association of OVVF with another anatomoclinical entity of fistula (AOR = 3.16; 95% CI:1.09–9.13), the fistula size > 3 cm (AOR = 3.65; 95% CI:1.36–9.76), the peri-operative hemorrhage (AOR = 7.01; 95% CI:2.33–21.03), and the post-operative infection (AOR = 178.89; 95% CI:26.09–1226.64). A score ranging from 0 to 13 points was obtained, of which a value ≤ 5 points defines a low risk of FSROVVF, a value between 6 and 8 points defines a moderate risk and value ≥ 9 points corresponds to a high risk of FSROVVF. The area under the ROC curve of the score is 0.925 with a sensitivity of 61.11%, a specificity of 96.59%, a positive predictive value of 78.57% and a negative predictive value of 92.39%.

**Conclusion:**

This study demonstrated that the number of fistulas ≥ 2, fistula size > 3 cm, fibrosis, association of OVVF with other types of fistulas, peri-operative hemorrhage, and post-operative infection are factors predictive of FSROVVF. These six factors are key contributors to the score used to predict FSROVVF. Once validated, this score will inform and enable preoperative counseling regarding the prognosis and the chances of a successful outcome of surgical repair of OVVF.

## Introduction

Obstetric vesico-vaginal fistula (OVVF) is a serious complication of childbirth that can have devastating physical, psychological and social consequences in the women who suffer from it [1]. It usually occurs as a result of prolonged obstructed labor in a resource-limited setting, where access to emergency obstetric care is often restricted. In addition to constant urinary incontinence, women with vesico-vaginal fistula (VVF) present with recurrent urinary tract infections and other medical complications [2].

Although surgical repair of VVF is the main treatment option, it can sometimes fail. Surgical repair of uro-genital obstetric fistulas aims to restore the anatomical and functional integrity of the urinary tract, enabling affected women to regain their dignity and quality of life [1, 3]. This complex procedure requires specialized medical expertise, as well as a suitable environment and adequate resources.

The success rates of VVF repairs vary between studies [4]. However, multiple lesion combinations and the absence of a standardized system of terminology, classification, data collection and reporting have made it difficult to assess and compare surgical outcomes [5]. In Pakistan (South Asia), the failure rate of surgical repair of VVF has been reported at 12.8% [6]. In sub-Saharan Africa, this rate varies from 12.9% in the DRC [7] to 58% in Angola [8]. An Ethiopian study reported a VVF surgical repair failure rate of 15.5% [9].

Numerous studies have attempted to determine the patient and fistula characteristics affecting the outcome of VVF surgery. Reported factors predictive of failure of surgical repair of VVF include large fistula size [9, 10], the presence of 2 or more fistulas in the same patient [4], history of repair [10], urethral lesions [4, 6], scar fibrosis [4, 9, 10], Goh type 3 or 4 fistula [9, 10], surgeon experience [11], abdominal repair [4, 9] and post-operative infection [10].

Determining the rate and identifying risk factors for failure of surgical repair of obstetric vesico-vaginal fistulas (FSROVVF) in participants undergoing fistula repair will better inform both participants and healthcare providers about relative risks, guide surgical approaches and ultimately improve the overall outcome of fistula repair in the DRC. The aim of this study was to determine the rate and identify predictors of fistula repair in order to develop a predictive score for fistula repair adapted to our setting.

## Materials and methods

### Type, period, and study population

This was an analytical cross-sectional study conducted from January 2017 to December 2022 in general referral hospitals in seven provinces of the DRC: HEAL Africa Hospital in Goma and General Referral Hospital (GRH) Beni (North Kivu province), GRH Wamba (Haut-Uélé province), GRH Lukonga (Central Kasai province), GRH Dr Amu-Yasa-Bonga (Kwilu province), GRH Kipaka and GRH Kasongo (Maniema province), GRH Karawa (North-Ubangi province) and GRH Katakokombe (Sankuru province). GRHs were selected to host surgical campaigns because they had the minimum infrastructures, had enough space for participants and had enough human resources to help with the feasibility of specialized surgeries such as fistula repairs. Each of these hospitals receives participants from nearby health centers. The above 9 hospitals we worked in, are located in 7 provinces out of the 26 that DRC has. Probably there are fistula women in almost all the 26 provinces of the DRC. To decide on where to go for surgeries, we considered provinces in which enough fistula women were identified and could be gathered at one place and be worked on from one big (referral hospital) hospital.

Participant awareness and recruitment was the result of obstetric fistula surgery campaigns organized by the non-governmental organization HEAL Africa in collaboration with the DRC’s national Ministry of Public Health, with the objective to provide women with access to quality and cost-free surgical fistula care. We included any participant with a VVF of obstetric origin seen as an outpatient or referred for surgical management during the study period. All participants received care from the same specialized surgical team, comprising an Obstetrician-Gynecologist and fistula surgeon, a general practitioner, a nurse with long experience in uro-gynecological care, an operating room nurse technician and a nurse anesthetist. In the operating room, after spinal anaesthesia was given and antibiotic prophylaxis performed, the participant was placed in an exaggerated lithotomy position, with her legs well over the edge of the operating table and in an accentuated Trendelenburg position. Surgical repair of the fistula involved the following steps, respectively: exposure of the fistula, catheterization of the ureters, especially when they were located close to the fistula margins; infiltration of the vesico-vaginal wall all around the fistula with mixed hemostatic solution (adrenaline, lidocaine, physiological solution) to help open the planes and facilitate dissection; incisions around the fistula through the vaginal epithelium; dissection of the vesico-vaginal wall for good and wide mobilization of the bladder; tension-free suturing of the bladder in two layers, very rarely in one layer when local conditions were poor (particularly in cases of severe scar fibrosis); performance of a dye test; tension-free suturing of the vaginal skin/mucosa. At the end of the repair, a Foley catheter was inserted with balloon inflation. A gauze pad was inserted into the vagina, to be removed the day after the operation.

A total of 318 OVVF women were eligible for treatment and recruited into the study.

### Study variables

Data were collected from participants’ files and operating room registries in each hospital.

We collected participants’ socio-demographic characteristics, which included age at the time of surgical repair (< 20 years, 20–29 years, 30–39 years, or ≥ 40 years), residence (rural or urban), and level of education (none, primary or secondary).

We also investigated participants’ parity at the time of surgical repair (1 or ≥ 2), place of delivery at the time of fistula occurrence (home or health facility), history of use of intravaginal indigenous products by participants as part of traditional fistula treatment in the community.

For clinical characteristics of the fistula, we collected the age of the fistula (< 1 year, 1–5 years, or > 5 years), the number of previous surgical repair attempts (none or ≥ 1), the number of vesico-vaginal fistulas in the same woman (1 or ≥ 2), the association of VVF with another type of fistula (yes or no), the urethral status (intact or partially/totally affected), the presence of scar fibrosis (yes or no), intra- and post-operative complications (none, peri-operative hemorrhage, or post-operative infection), and the fistula size (≤ 3 cm or > 3 cm).

We also recorded the results of the surgical repair (failure or success) as documented by the final physical examination at the time participants were discharged home. Participants were divided into two groups according to the outcome of their surgical repair. The outcome was defined as follows:

- Failure of surgical repair defined as non-closure of the fistula. In these cases, the fistula was not fully closed, even though urine leakage often diminished considerably with or without continued micturition.
- Successful surgical repair defined as closure of the fistula. In these cases, the fistula was completely closed, with or without urinary incontinence. There was no leakage of urine at the fistula site.

### Statistical analysis

Data were double-entered on separate Excel sheets, based on participant records, operation notes and operating room registries from hospitals that had hosted the mobile surgery campaigns. Data base was checked, errors were corrected, and missing data were filled from the participants records and operating theatre registries. A single database was obtained and then transferred from Microsoft Excel to STATA 16 for statistical analysis.

Statistical analyses were performed using STATA 16 software. Data from participants with failed surgical repair of OVVF were compared with those whose surgical repair had been successful.

These analyses examined the various contributing factors or explanatory variables (independent variables) one by one, to search for any significant association with FSROVVF (dependent variable). The association between an explanatory variable and FSROVVF was measured by calculating odds ratios and their 95% confidence intervals. Pearson’s Chi-square test was used to compare observed proportions. Statistical significance was set at *p* < 0.05.

All variables with a significance level of less than 0.2 in the unifactorial analysis were included in a multivariate analysis using logistic regression.

To build the multivariate model, we opted for the mixed stepwise selection method at the *p* < 0.05 threshold. The logistic model thus made it possible to analyze the contribution of each explanatory variable to the FSROVVF in the presence of the other independent variables, and not the participation of the explanatory variables taken in isolation.

The discrimination of the logistic model was assessed by calculating the area under the Receiver Operating Characteristics (ROC) curve. The graphical expression of score discrimination is the ROC curve, which plots sensitivity values against the complement of specificity (1- specificity). The score was calibrated using the Hosmer–Lemeshow test [12]. The score’s discrimination is its ability to separate subjects with and without disease [13]. Sensitivity, specificity and the percentage of correctly classified cases were then determined in relation to the c statistic. The robustness of the model’s coefficients was assessed by bootstrapping.

A predictive risk score was derived from the statistical analysis. In order to develop a screening tool to predict FSROVVF, points were assigned to each risk factor retained in the logistic model. To make it simple and usable, the score was estimated using the rounded values of these coefficients [14]. The risk probabilities of FSROVVF according to the values of the constructed score were also calculated.

The study was approved by the Medical Ethics Committee of the University of Goma (Approval No.: UNIGOM/CEM/011/2022). The data was collected anonymously. The study did not present any direct monetary benefits for the study participants.

## Results

A total of 318 participants with OVVF underwent surgical repair. Surgical repair was unsuccessful in 16.98% of cases (54/318).

Table 1 shows that there was no statistically significant association between FSROVVF and socio-demographic characteristics such as participant age at intake, parity at intake, residence, level of education and place of delivery (*p* > 0.05). On the other hand, a statistically significant association was found between FSROVVF and the use of intravaginal indigenous products (Crude OR = 2.45 [1.30–4.61]; *p* = 0.0073).

**Table 1:** Sociodemographic characteristics and history correlated with failure of surgical repair among women with obstetric vesico-vaginal fistula in the Democratic Republic of the Congo (*N* = 318)

Variable	**Result of surgical repair of obstetric vesico-vaginal Fistula**				**Total** **(*N* = 318)**	**Crude odds ratio [95% confidence interval]**	***p*-value**
	**Failure (*n* = 54)**		**Success (*n* = 264)**				
**Age at repair**							
20 years	6	25.00%	18	75.00%	24	1.00	
20–29 years	15	16.00%	79	84.00%	94	0.57 [0.19–1.67]	0.4624
30–39 years	21	21.40%	77	78.60%	98	0.82 [0.29–2.32]	0.9176
≥ 40 years	12	11.80%	90	88.20%	102	0.40 [0.13–1.20]	0.1792
**Residence**							
Rural	43	17.84%	198	82.16%	241	1.30 [0.63–2.67]	0.5828
Urban	11	14.29%	66	85.71%	77	1.00	
**Education level**							
None	21	16.80%	104	83.20%	125	1.00	
Primary	17	13.18%	112	86.82%	129	0.75 [0.38–1.50]	0.5267
Secondary	16	25.0%	48	75.0%	64	1.65 [0.79–3.45]	0.2498
**Place of delivery**							
Home	20	16.81%	99	83.19%	119	1.00	
Health facilities	34	17.09%	165	82.91%	199	1.02 [0.55–1.87]	1.0000
**Parity at repair**							
1	33	18.00%	150	82.00%	183	1.19 [0.66–2.17]	0.6669
≥ 2	21	15.56%	114	84.44%	135	1.00	
**Use of intravaginal indigenous products**							
No	16	10.67%	134	89.33%	150	1.00	
Yes	38	22.62%	130	77.38%	168	2.45 [1.30–4.61]	0.0073

As for clinical characteristics, Table 2 shows that fistula age and previous fistula repair attempt were not significantly associated with FSROVVF (*p* > 0.05). We did, however, find a significant association between FSROVVF and the number of fistulas, association of VVF with another type of fistula, urethral condition, scar fibrosis, fistula size and post-operative complications.

**Table 2:** Clinical features correlating with failure of surgical repair in women with obstetric vesico-vaginal fistula in the Democratic Republic of the Congo (*N* = 318)

Variable	**Result of surgical repair of obstetric vesico-vaginal Fistula**				**Total** **(*N* = 318)**	**Crude odds ratio [95% confidence interval]**	***p*-value**
	**Failure (*n* = 54)**		**Success (*n* = 264)**				
**Fistula age at repair**							
< 1 year	10	20.41%	39	79.59%	49	1.42 [0.63–3.17]	0.5217
1–5 years	16	18.60%	70	81.40%	86	1.26 [0.64–2.49]	0.6125
> 5 years	28	15.30%	155	84.70%	183	1.00	
**Previous repair attempt**							
No	25	14.79%	144	85.21%	169	1.00	
≥ 1	29	19.46%	120	80.54%	149	1.39 [0.77–2.50]	0.3385
**Number of vesico-vaginal fistula in the same participant**							
1	12	6.19%	182	93.81%	194	1.00	
≥ 2	42	33.87%	82	66.13%	124	7.77 [3.89–15.53]	< 0.0001
**Association of Vesico-Vaginal Fistula with other types of fistula**							
No	30	13.10%	199	86.90%	229	1.00	
Yes	24	26.97%	65	73.03%	89	2.45 [1.34–4.49]	0.0052
**Condition of the urethra**							
Intact	46	15.65%	248	84.35%	294	1.00	
Partial damage	4	22.22%	14	77.78%	18	1.54 [0.35–5.19]	0.5047
Total damage	4	66.67%	2	33.33%	6	10.65 [1.92–60.60]	0.0080
**Presence of scarring fibrosis**							
No	12	7.02%	159	92.98%	171	1.00	
Yes	42	28.57%	105	71.43%	147	5.30 [2.67–10.54]	< 0.0001
**Post-operative complications**							
No	24	8.73%	251	91.27%	275	1.00	
Perioperative Hemorrhage	11	50.00%	11	50.00%	22	10.46 [4.11–26.63]	< 0.0001
Post-operative infection	19	90.48%	2	9.52%	21	96.02 [21.24–899.15]	< 0.0001
**Size of fistula**							
< 1.5 cm	2	4.44%	43	95.56%	45	1.00	
1.5–3 cm	7	5.83%	113	94.17%	120	1.33 [0.24–13.61]	1.0000
> 3 cm	45	29.41%	108	70.59%	153	8.96 [2.15–78.95]	0.0011

FSROVVF was greater in the presence of two or more fistulas (Crude OR = 7.77; 95% CI: 3.89–15.53; *p* < 0.0001), in the presence of another type of fistula (Crude OR = 2.45; 95% CI: 1.34–4.49; *p* < 0.0052), when the urethra was totally involved (Crude OR = 10.65; 95% CI: 1.92–60.60; *p* = 0.0080), in the presence of scar fibrosis (Crude OR = 5.30; 95% CI: 2.67–10.54; *p* < 0.0001), when the fistula measured 3 cm or more (Crude OR = 8.96; 95% CI: 2.15–78.95; *p* = 0.0011), in the presence of perioperative hemorrhage (Crude OR = 10.46; 95% CI: 4.11–26.63; *p* < 0.0001) and in the presence of postoperative infection (Crude OR = 96.02; 95% CI: 21.24–899.15; *p* < 0.0001).

After logistic regression, six criteria emerged as predictive factors for FSROVVF (Table 3):

- ◦ Presence of scar fibrosis (AOR = 5.01; 95% CI: 1.73–14.49; *p* = 0.003);
- ◦ Presence of 2 or more VVFs in the same participant (AOR = 9.04; 95% CI: 3.01–27.13; *p* < 0.0001);
- ◦ Association of a VVF with another type of fistula (AOR = 3.16; 95% CI: 1.09–9.13; *p* = 0.034);
- ◦ Fistula size > 3 cm (AOR = 3.65; 95% CI: 1.36–9.76; *p* = 0.010);
- ◦ Peri-operative hemorrhage (AOR = 7.01; 95% CI: 2.33–21.03; *p* = 0.001);
- ◦ Post-operative infection (AOR = 178.89; 95% CI: 26.09–1226.64; *p* < 0.0001).

**Table 3:** Logistic regression model coefficients and scores of the risk of FSROVVF

Variable	**Adjusted odds ratio** **[95% confidence interval]**	**Coefficient**	***p*-value**	**Score**
Scarring fibrosis	5.01 [1.73–14.49]	1.61	0.0030	2
Number of fistulas ≥ 2	9.04 [3.01–27.13]	2.20	0.0001	2
Fistula size > 3 cm	3.65 [1.36–9.76]	1.29	0.0100	1
Association of vesico-vaginal fistula with other types of fistula	3.16 [1.09–9.13]	1.15	0.0340	1
Perioperative hemorrhage	7.01 [2.33–21.03]	1.95	0.0005	2
Post-operative infection	178.89 [26.09–1226.64]	5.19	< 0.0001	5

The predictive score for FSROVVF was constructed using the logistic model. Each risk factor was weighted by a regression coefficient representing the weight of the variable in the calculation of the score, with all the scores obtained illustrated below (Table 3).

**Fig. 1:**
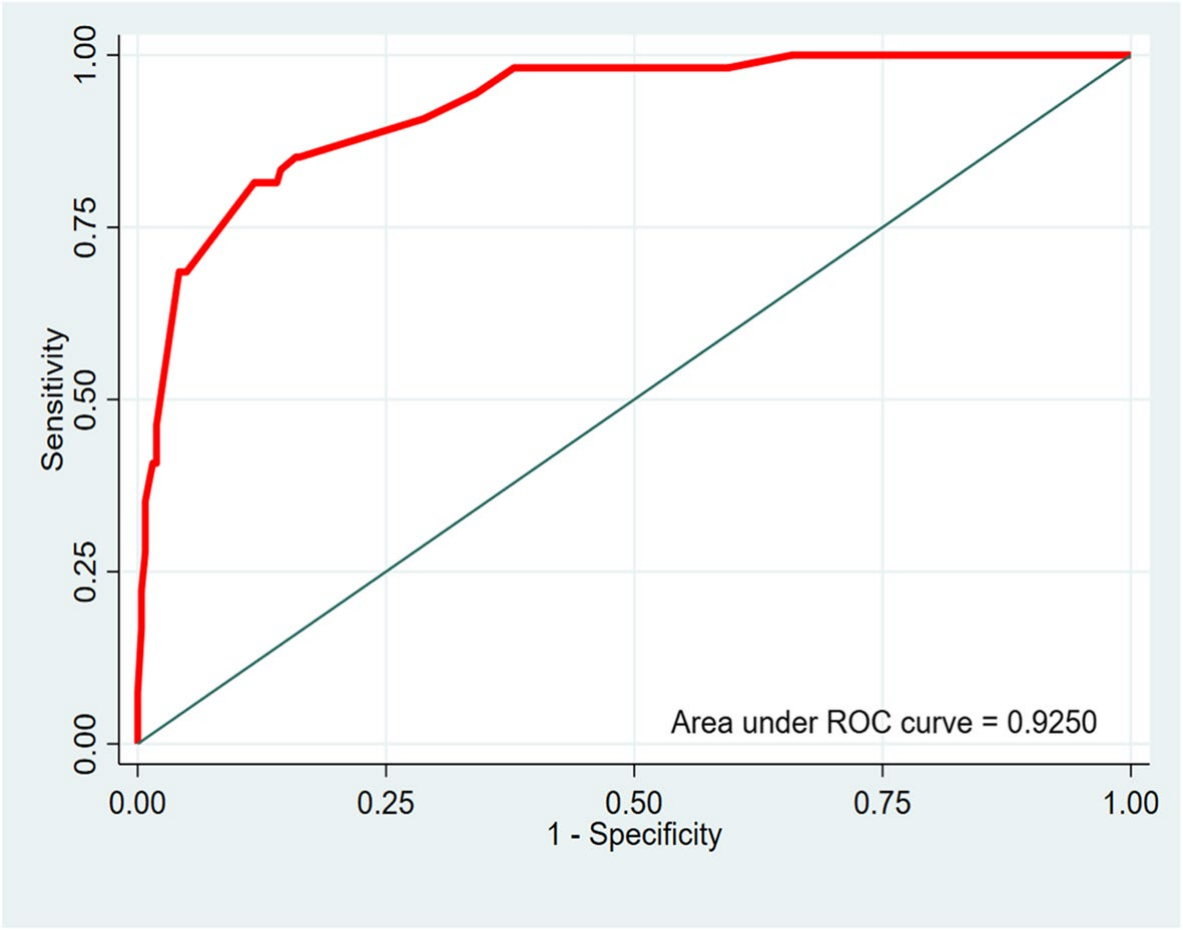
ROC Curve of FSROVVF predictive score

The area under the ROC curve of the score is 0.925 (Fig. 1). This curve shows excellent discrimination in terms of its ability to distinguish between participants who will end with FSROVVF and those who will not.

The presence of these six criteria corresponds to a certain number of points, totaling 13. For each participant, the score varies from 0 to 13, and the higher the score, the higher the risk of FSROVVF.

The probabilities of risk of FSROVVF according to the values of the constructed score have been calculated and are presented in Table 4.

**Table 4:** Probability of FSROVVF by score according to logistic regression model

Score obtained	Probability of failure*
0	0.22%
1	0.54%
2	1.35%
3	3.34%
4	8.02%
5	18.02%
6	35.66%
7	58.30%
8	77.90%
9	89.89%
10	95.73%
11	98.26%
12	99.30%
13	99.72%

^*^Obtained from the formula:

*p* = *1/1* + *exp (-6.14 – 0.9250* × *score)*

A score ≤ 5 points defines a group of participants at low risk of FSROVVF; a score between 6 and 8 points defines a moderate risk of FSROVVF and a score ≥ 9 points presents a high risk of FSROVVF.

Thus, for this score predictive of FSROVVF, a sensitivity of 61.11% was obtained for a specificity of 96.59%. The positive predictive value was 78.57% and the negative predictive value 92.39%.

## Discussion

Vesico-vaginal fistula (VVF) is a devastating condition that causes physical, psychological and social disability for affected women, particularly in low-income countries [15]. Surgical repair is the primary treatment for this condition. Success rates vary according to the skill and experience of the surgeon, as well as the complexity of the fistula. In the present study, the success rate of surgical repair of OVVF was 83.02%. This success rate is close to the 85% set by the WHO to determine the level of quality of services offered to participants in a fistula treatment center [16]. A study conducted in Nord-Ubangi Province (in the DRC) by Paluku et al. reported an overall success rate of 87.1% for 163 consecutive repairs of OVVF [7]. Similarly, a retrospective analysis of 384 Congolese women undergoing surgical repair of VVF in Haut-Katanga Province (in the DRC) by Nsambi et al. [4] revealed an overall success rate of 82.8%. Similar high success rates were reported in several studies ranging from 84.3% to 94% [9, 17–21].

These differences in success rates could be explained by the clinical difference of the fistulas treated, the surgeons’ experience in fistula repair, the technical platforms used, the selection of cases of fistulas to be repaired, the choice of approach for the different cases of fistula repaired, and even the definition of surgical success, which differ from study to study.

Risk factor analysis for FSROVVF was based on comparison of women with FSROVVF and women without FSROVVF. Our results provide stronger evidence of the negative impact of pre-operative clinical fistula characteristics on surgical repair outcomes, as opposed to participant characteristics. In the present study, FSROVVF was significantly associated with scar fibrosis, the presence of two or more fistulas, fistula size > 3 cm, the presence of peri-operative hemorrhage and that of post-operative infection.

Our study found that scar fibrosis is a potential risk factor for repair failure. Several studies have demonstrated that the presence of scar fibrosis can reduce the chances of successful surgical repair of VVF [4, 18, 22, 23]. In a study by Nardos et al. [22], involving 268 participants undergoing surgical repair for OVVF, the presence of moderate to severe scar fibrosis was associated with a repair failure rate of 17%, compared with only 3% in participants with no scar fibrosis or mild fibrosis (AOR = 2.67; 95% CI: 1.58–4.50), concluding that scar fibrosis is an independent risk factor for VVF repair failure. A case–control study of risk factors associated with OVVF carried out on 420 women by Barageine et al. [24] in Uganda revealed that participants with severe scar fibrosis were 3.5 times more likely to suffer repair failure than those without scar fibrosis (AOR = 3.5; 95% CI: 1.6–7.6). Similarly, a study by Nsambi et al. [4] in Haut-Katanga province (in the DRC) reported that participants with scarring fibrosis were 15 times more likely to undergo repair failure than those without scarring fibrosis (AOR = 15.22; 95% CI: 7.34–31.58).

Scar fibrosis is a pathological process characterized by excessive scar tissue formation during lesion healing. Fibrosis can compromise the success of surgical repair of VVF in several ways. First, the presence of scar tissue can make dissection and closure of the fistula more difficult, increasing the chances of intraoperative complications such as bleeding and damage to surrounding structures. Second, fibrosis can restrict the mobility of surrounding tissues, which can lead to excessive tension on the suture and subsequent dehiscence of the repair [5, 18]. In addition, fibrous scarring can impair local vascularization, compromising normal healing and suture consolidation. Fibrosis can complicate surgical repair by making tissues less supple and more difficult to suture. Moreover, the presence of fibrosis can also increase the risk of fistula recurrence after surgery. It is therefore crucial for surgeons to consider the presence of scarring fibrosis when planning and performing surgical repair of vesico-vaginal fistula [22].

The presence of multiple fistulas may represent an additional challenge for surgical repair. The present study showed that the presence of two or more OVVFs in the same participant (AOR = 9.04; 95% CI: 3.01–27.13) and the association of an OVVF with other anatomoclinical fistula entities (AOR = 3.16; 95% CI: 1.09–9.13) were reported as significant predictors of FSROVVF. The Congolese study by Nsambi et al. [4] revealed that participants with two or more fistulas had a repair failure rate of 58.33% versus 12.54% in those with a single fistula (AOR = 7.41; 95% CI: 3.05—17.97), suggesting that the presence of multiple fistulas was an independent risk factor for failure of surgical repair of VVF. These authors point out that the presence of several fistulas in the same participant makes local tissue mobilization difficult and does not allow repair under tension due to the scarcity of tissue in the bladder [4].

For this reason, several authors suggest suturing the bladder and vagina separately without tension after separation of the vaginal plane from the bladder plane around the fistula. Wide separation of the two planes allows sufficient mobilization of the bladder  and permits tension-free suturing [4, 25]. The presence of multiple fistulas can lead to increased complexity of the surgical procedure, with additional difficulties associated with dissection, fistula closure and reconstruction of surrounding tissue. Furthermore, the presence of multiple fistulas may compromise local vascularization and increase the risk of tissue adhesions, which may lead to failure of surgical repair [18].

 Previous studies [4, 18, 25] support the idea that fistula size > 3 cm is an independent risk factor for failure of surgical repair of VVF. In our study, participants with VVFs > 3 cm in size were more than 3 times, more likely to have failed repair than those with fistulas ≤ 3 cm in size (AOR = 3.65; 95% CI: 1.36–9.76).

This finding is identical to that of a Ugandan study by Kayondo et al. (OR = 6; 95% CI: 1.46–24.63) [5]. An Ethiopian study by Meikena et al. [26], which included 328 VVF participants, revealed a surgical repair failure rate of 3.5% for participants with fistula size ≤ 3 cm, while it was high at 15.6% for participants with fistula size > 3 cm (AOR = 11.68; 95% CI: 1.41–96.42). A larger fistula size can make the surgical procedure more complex, with additional challenges such as tissue manipulation, fistula closure and reconstruction of surrounding structures. In addition, larger fistulas may be associated with more extensive tissue loss and impaired vascularization, compromising successful repair [5]. Larger fistulas often pose greater challenges during surgical repair.

Closure often requires more complex techniques, including mobilization of surrounding tissue, skin or mucosa grafts, or even combined approaches. Poor vascularization of surrounding tissue can make it difficult for tissue grafts to heal and survive. In addition, excessive tension on the fistula closure can lead to tissue necrosis and dehiscence.

The present study found that participants with post-operative infection were more than 179 times more likely to have a FSROVVF (AOR = 178.89; 95% CI: 26.09–1226.64). Post-operative infection is a factor that has a significant negative impact on the outcome of surgical fistula repair, as also reported by Aynie et al. [10]. According to the Ethiopian study by Meikena et al. [26], participants who received antibiotic prophylaxis for 7 days in the peri-operative period had a repair failure rate of 8.2%, compared with 19.4% in those who did not receive prophylactic antibiotics, indicating that prevention of post-operative infection by antibiotics was a protective factor against FSROVVF (AOR = 0.125; 95% CI: 0.028–0.555).

Post-operative infections can compromise tissue healing and increase the risk of fistula recurrence. Post-operative infections can lead to complications such as increased suture dehiscence, delayed healing, pus accumulation and tissue inflammation, compromising fistula healing. In addition, infections can lead to further complications, such as abscess formation or sepsis, which can be life-threatening and require further medical intervention. To minimize the risk of post-operative infection and improve the outcome of surgical repair, it is essential to follow strict infection prevention protocols, such as administering prophylactic antibiotics, maintaining a sterile environment and careful monitoring for signs of infection posoperatively. Furthermore, a randomized controlled trial conducted in Benin concluded that women in the antibiotic prophylaxis group received fewer post-operative antibiotics and had fewer urinary tract infections, but that antibiotic prophylaxis did not reduce the chances of failed repair (OR = 2.1; 95% CI: 0.75–6.1) [27].

This study found a significant association between FSROVVF and peri-operative hemorrhage. Our results provide further evidence to support the role of peri-operative hemorrhage in predicting FSROVVF. Women with peri-operative hemorrhage were 7 times more likely not to have fistula closure than those without peri-operative hemorrhage (AOR = 7.01; 95% CI: 2.33–21.03). A recent Ugandan study by Holt et al. [28] reported that the rate of repair failure was significantly higher when a participant received peri-operative transfusion (AOR = 3.10; 95% CI: 1.11–8.66). The results of these studies highlight the negative impact of peri-operative hemorrhage on the outcome of surgical repair of VVF.

Hemorrhage can lead to reduced surgical visibility, difficulty in achieving accurate dissection and impaired suture quality. In addition, hemorrhage can increase the risk of post-operative complications such as infection and hematoma formation, which can compromise fistula healing. To minimize the risk of peri-operative hemorrhage and improve the outcome of surgical repair, it is essential to follow appropriate surgical protocols, such as mastering dissection and suturing techniques, using suitable surgical instruments and carefully monitoring for signs of hemorrhage during and after surgery.

FSROVVF is a major risk factor for poor participant quality of life. The ability to predict FSROVVF has always been the main concern of urologists and gynecologists repairing VVF. Accurate prediction of cases at risk of FSROVVF would make it possible to select cases meriting a higher standard of care, or more often, in our environment, early transfer to hospitals specializing in the management of VVF.

Thus, prediction of FSROVVF in at-risk participants must be sufficiently specific to avoid unnecessary referral. Hence the need for a predictive score to guide screening and the eventual decision-making process on where participants should undergo surgery. This means that decisions based on the scoring of score parameters using easy-to-collect clinical variables, as proposed in this article, can help avoid FSROVVF and repeat surgery.

Our study shows that the clinical score obtained by consolidation and scoring these six parameters proved to be the best model for identifying participants at risk of FSROVVF. Although moderately sensitive (61.11%), this score has a very high specificity (96.59%) and will therefore enable better detection of participants at risk of FSROVVF and seek to optimize surgical outcome (by referral to specialized centers). The advantage of the proposed score is that the clinical variables used can easily be identified during a routine pre-operative visit. 

This score, after being validated in other populations, could be very useful in peripheral hospitals where participants with OVVF are recruited, to identify fistula women at risk of surgical repair failure and refer them to specialized referral hospitals for surgical care. Preventing FSROVVF thus means avoiding repeated and traumatic surgical interventions for participants, since the best chance for success is the initial attempt at repair. Every effort should be made to attain a successful result the first time, because each subsequent operation would create further sclerosis, making future attempts more difficult.

The strength of the present study is that it included a large number of participants with similar pathology in several provinces of the DRC. All participants benefited from the services of the same surgical team, with same expertise and using identical equipment. The same variables were taken from each participant and recorded.

However, as a limitation of this study, it is important to note that participants who remained incontinent at hospital discharge despite complete closure of their fistulas were classified as failures. It cannot be excluded that some of them became continent within weeks or months following surgery. The use of urodynamics during the discharge assessment and the review of these participants a few months after surgery would have made it possible to identify them and classify them as cured, thus increasing the success rate.

## Conclusion

The present study revealed that in the DRC, the failure rate of surgical repair of obstetric vesico-vaginal fistula is less than twenty-five percent of all treated participants. The study developed a score to predict the risk of surgical repair failure, with factors including the number of fistulas greater than or equal to two, fistula size greater than three centimeters, presence of fibrosis (scarring), association with other fistulas, peri-operative haemorrhage and post-operative infection. There is a need to develop a more advanced therapeutic approach that takes into account such risk factors as predictive indicators for failed surgical repair. Once validated, the predictive score will help implement a pre-operative participant counselling and surgical management regimen tailored to each case of obstetric vesico-vaginal fistula. All this will help improve the outcome of surgical management of obstetric vesico-vaginal fistulas in the DRC and ultimate well-being of fistula women.

## Data Availability

The datasets used and/or analyzed during the current study are available from the corresponding author on reasonable request.

## Abbreviations

95% CI: 95% Confidence interval
AOR: Adjusted odds ratio
DRC: Democratic Republic of the Congo
FSROVVF: Failure of surgical repair of obstetric vesico-vaginal fistula
GRH: General referral hospital
OF: Obstetric fistula
OR: Odds ratio
OVVF: Obstetric vesico-vaginal fistula
VVF: Vesico-vaginal fistula
